# Third-Phase Formation in Rare Earth Element Extraction with D2EHPA: Key Factors and Impact on Liquid Membrane Extraction Performance

**DOI:** 10.3390/membranes15070188

**Published:** 2025-06-23

**Authors:** Raquel Rodríguez Varela, Alexandre Chagnes, Kerstin Forsberg

**Affiliations:** 1 KTH Royal Institute of Technology, Department of Chemical Engineering, Teknikringen 42, 114 28 Stockholm, Sweden; raquelrv@kth.se; 2 GeoRessources, Université de Lorraine, CNRS, 54000 Nancy, France; alexandre.chagnes@univ-lorraine.fr

**Keywords:** third-phase formation, rare earth elements, D2EHPA, limiting organic concentration (LOC), hollow fibre renewal liquid membrane (HFRLM)

## Abstract

Hollow fibre renewal liquid membranes (HFRLMs) are susceptible to third-phase formation during rare earth element (REE) extraction using D2EHPA (bis(2-ethylhexyl phosphoric acid)), potentially leading to membrane fouling and decreased mass transfer efficiency. This study investigated the effects of various parameters, such as the composition of the aqueous feed and organic phases, on the third-phase formation and limiting organic concentration (LOC) of REE(III) in D2EHPA. Higher concentrations of REEs and a higher pH in the feed phase correlated with decreased mass transfer, while yttrium showed a greater propensity to induce third-phase formation compared to other REEs. Conditions favouring the use of linear aliphatic diluents, low extractant concentrations (5–10 *v*/*v*% D2EHPA) and the absence of modifiers also contributed to third-phase formation. The addition of tri-n-butyl phosphate (TBP) mitigated third-phase formation without evidence of synergy with D2EHPA. These findings provide key insights into formulating extraction systems that prevent third-phase formation in HFRLM processes.

## 1. Introduction

Rare earth elements (REEs) have numerous applications in both traditional and high-tech sectors due to their unique physicochemical properties [1]. These elements, which include the 15 elements with atomic numbers 57 to 71 (from lanthanum to lutetium) plus yttrium and scandium, have been identified as critical elements with a high import reliance and an impossibility of being substituted by other elements without the loss of performance [2].

The industrial extraction of REEs is predominantly performed by solvent extraction [1,3]. Despite the widespread use of this technique, it can involve challenges. These include the need for a density difference between the phases, emulsification problems, flooding and loading limitations and high capital and operating costs [4]. In this context, hollow fibre liquid membranes (HFLMs) have emerged as a promising alternative for certain systems that can overcome these limitations. Furthermore, due to the large surface area per volume of the fibres, HFLMs can achieve a defined extraction/separation faster than conventional processes [5].

Among the various HFLM configurations, the hollow fibre renewal liquid membrane (HFRLM) technique has shown great potential for the separation of solutes due to its high membrane stability [6]. A summary of previous studies on metal extraction by means of HFRLMs is presented in Table 1. In the HFRLM technique, the hydrophobic polymeric hollow fibre structure is impregnated with the organic phase constituted by a selective extractant and a diluent. The organic phase is held within the pores by capillary forces, thereby forming the liquid organic membrane. One of the aqueous phases is pumped through the shell side of the fibres, while a dispersion of the other aqueous phase and some of the organic phase with a high aqueous-to-organic ratio (A:O) is pumped through the lumen side of the fibres. This dispersion enables the replenishment of the liquid organic membrane that is eventually removed from the pores of the support by shear forces [7,8]. Furthermore, the aqueous solution becomes saturated with the organic compounds, preventing dissolution from the organic phase in the pores. Despite the advantages of this technique, its lower organic inventory and the difficulties in ensuring the complete cleaning of the fibres could potentially promote third-phase formation in the organic phase. The formation of a third phase would result in a reduction in mass transfer due to the partial or total fouling of the hollow fibre pores and the reduced availability of extractant molecules. Consequently, this would result in a reduction in the permeate flux and even a potential cessation of the solute extraction. Despite the significant impact of the formation of a third phase on the performance of HFRLMs, this phenomenon is not commonly evaluated for this type of application.

### 1.1. The Mechanism Behind Third-Phase Formation

**Figure 1:**
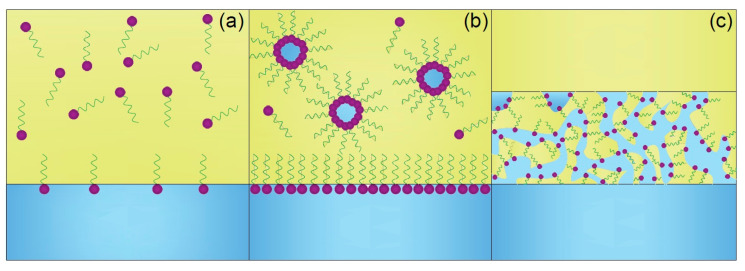
Third-phase formation mechanism. (**a**) Adsorption of extractant at the aqueous–organic interphase; (**b**) formation of reverse micelles after the saturation of the interphase; (**c**) third-phase formation after LOC is reached. The organic phase is represented in yellow and the aqueous phase is represented in blue.

Extractants used in liquid–liquid extractions are typically formed of a polar head group, which is reactive towards the solute, and a hydrophobic tail [25]. When extractant molecules adsorb at the aqueous–organic interphase, the hydrophilic head group extends into the aqueous phase while the hydrophobic alkyl chain extends into the organic phase (Figure 1a). When the interphase is nearly saturated with extractant molecules, the extractant begins to aggregate into reverse micelles in the organic phase to minimise the free energy of the system (Figure 1b) [25,26]. The aggregates formed interact with each other due to three physical forces: van der Waals attraction between polar water molecules in oil, infinite repulsion between hard cores and steric repulsion between chains of diluent and extractant. In addition, extractants can solubilise polar compounds such as water or extractant–solute complexes, which significantly increases the size of the reversed micelles and affects molecular interactions [25,27,28].

As the solute concentration in the organic phase increases, the resulting forces can lead to demixing when the attractive intermicellar forces exceed two times the thermal energy. This phenomenon is the origin of the formation of the third phase in liquid–liquid extraction [29], where the organic phase splits into two phases (Figure 1c). The upper organic phase consists mainly of the organic diluent, whereas the middle organic phase, or third phase, is primarily constituted by the extractant and the extracted complexes [26]. The maximum solute concentration that can be solubilised in the organic phase before the third-phase formation is the limiting organic concentration (LOC) [30,31].

Consequently, the aggregation behaviour of the extractant under specific operating conditions determines phase instability [32] and influences several key parameters, including diffusion rate, extraction mechanism [33], reaction kinetics and species solubility. However, the intermolecular aggregation of extractants in the organic phase is often overlooked despite its significant impact on extraction processes [34].

### 1.2. Third-Phase Formation in REE Extraction with D2EHPA

Bis(2-ethylhexyl)phosphate, also known as di(2-ethylhexyl)phosphoric acid and commonly referred to as D2EHPA, HDEHP, or P204, is one of the most commonly used extractants in REE extraction [35]. Despite its extensive use, there have been few studies on the aggregation and formation of third phases in systems involving D2EHPA [36].

Peppard et al. (1957) studied the LnCl_3_–D2EHPA–toluene system and found that an amorphous solid phase was formed in the low-proton-concentration region at a molar ratio Ln^3+^:D2EHPA of 1:6. The solid phase was identified as Ln(DEHP)_3_(HDEHP)_3_ or the mono-ionised dimeric form Ln[H(DEHP)_2_]_3_ [37].

Anticó et al. (1996) studied the YCl_3_–D2EHPA–kerosene system and showed that, in the D2EHPA concentration range from 0.10 to 0.35 M, aggregation began at intermediate Y(III) concentrations (210–2281 mg/L) and the deviation of the system from ideality increased with metal concentration. Third-phase formation at high metal concentrations (8000 mg/L) and 0.1 M H^+^ occurred at a Y(III):D2EHPA ratio between 1:2.2 and 1:9.9 and a Y:P ratio of 1:3, corresponding with the formation of the polymer (LnA_3_)_m_. The third phase was not formed at H^+^ feed concentrations of 1.0 M and 1.5 M. Comparable results were obtained for systems in chloride, perchlorate and nitrate media [38] and the aggregation process was described as follows [39]:

(1) $${Y\left(III\right)}_{aq}+{{n(HA)}_{2}}_{org}⇌{YA}_{3}\cdot {\left(2n-3\right)HA}_{org,3rdphase}+{{3H}^{+}}_{aq}$$

Harada and Smutz (1970) reported the formation of a precipitate with a Y(III):D2EHPA concentration ratio of 0.145 ± 0.005 and a Y(III)–phosphorous ratio of 1:3 in the system Y(NO_3_)_3_–D2EHPA–kerosene with 1M D2EHPA. The precipitate was of an irreversible nature and was not redissolved by the addition of excess acid [40]. The degree of polymerisation varied depending on the lanthanide ion involved and the process was described by the following equilibrium [41]:

(2) $${{nM}^{3+}}_{aq}+{n{M\left(HA\right)}_{3}}_{org}⇌{{\left({MA}_{3}\right)}_{n}}_{org,3rdphase}+{{3nH}^{+}}_{aq}$$

Doležal et al. (2000) investigated the conditions under which third-phase formation occurs in a flat sheet supported liquid membrane in the Y(NO_3_)_3_–D2EHPA–kerosene system. It was found that third-phase formation at the feed–membrane interphase was favoured by higher feed pH, higher extractant concentration in the organic phase and in the case of the extraction of lanthanide ions exhibiting higher atomic numbers [42].

Yurtov et al. (2006, 2007) studied the Tb(OH)_3_–D2EHPA–*n*-decane and Tb(NO_3_)_3_–D2EHPA–*n*-decane systems. In the hydroxide system, gel was formed in the bulk organic phase for D2EHPA:Tb molar ratios equal to or greater than 1.8, i.e., in the region where basic (mono- and disubstituted) bis(2-ethylhexyl) phosphates are formed. The gel was not thermodynamically stable and could be irreversibly destroyed by mechanical shear. At lower D2EHPA:Tb(OH)_3_ ratios, a precipitate was formed at the aqueous–organic interphase, or two separate liquid phases were formed. In the terbium nitrate system, a structural layer was formed at the aqueous–organic interphase. In all systems, the third phase was observed to start as an amorphous solid but to crystallise progressively over time [43,44].

Tomita et al. (2003) studied the SmCl_3_–D2EHPA–heptane and EuCl_3_–D2EHPA–n-heptane systems. Solid films appeared under similar conditions for the different systems, showing that third-phase formation was favoured by a high aqueous pH, high REE concentration in the feed phase and relatively low extractant concentration in the organic phase. However, the third phase was not formed at too low an extractant concentration ([(HR)_2_] < 5 × 10^−5^ M) and microemulsions were formed instead of a third phase at a higher extractant concentration ([(HR)_2_] > 0.5 M). REE extraction kinetics were significantly faster in the third-phase formation zone than in the low- and high-extractant-concentration regions. Third phases were found to correspond to the 1:3 polymeric complex containing one lanthanide ion and three extractant ligands [45].

The aim of this study is to identify the factors that influence third-phase formation during the extraction of REEs by D2EHPA in order to find an optimal chemical system composition that prevents the formation of the third phase in the HFRLM system. The effect of third-phase formation on mass transfer in the HFRLM system during the extraction of a multicomponent mixture of REEs by D2EHPA is evaluated. Furthermore, the effect of the composition of both the feed and the organic phase on the formation of the third phase is investigated by determining the limiting organic concentration (LOC). The chemical interactions in the organic phase are investigated by FT-IR.

## 2. Experimental Procedure

### 2.1. Reagents and Materials

The extractant di-(ethylhexyl) phosphoric acid (D2EHPA, CAS No. 298-07-7, 95 wt%) was supplied by Merck (Rahway, NJ, USA). The phase modifiers tri-n-butyl phosphate (TBP, CAS No. 126-73-8, ≥99 wt%) and 1-decanol (CAS No. 112-30-1, ≥98 wt%) were supplied by Acros Organics (Geel, Belgium). For the preparation of the organic phases, all chemicals were used without further purification and dissolved in the corresponding diluent to the desired concentration. The diluents used in this study were kerosene (CAS No. 64742-47-8) supplied by Alfa Aesar (Ward Hill, MA, USA), toluene (CAS No. 108-88-3, Merck), n-heptane (CAS No. 142-82-5) supplied by VWR (Radnor, PA, USA), cyclohexane (CAS No. 110-82-7, Merck) and n-dodecane (CAS No. 112-40-3) supplied by Sigma-Aldrich (Saint Paul, MO, USA).

Aqueous solutions of single and mixed REE solutions were prepared by dissolving La(III), Nd(III), Gd(III), Dy(III) and Y(III) oxides (99.9%), and Ce(III) carbonate (99.9%) supplied by Treibacher (Althofen, Austria), in hydrochloric acid diluted in deionised water. The pH values of aqueous phases were adjusted with hydrochloric acid and sodium hydroxide or ammonia solutions. The stripping phase was prepared by diluting definite amounts of nitric acid in deionised water.

The hollow fibre contactor used in this study was the Liqui-Cel Extra Flow 2.5 × 8 (Model G501; more information in Table 2) supplied by 3M (Saint Paul, MN, USA).

### 2.2. Analytical Methods

REE concentrations in the feed and stripping phases were measured using an ICP-OES spectrometer iCAP 4700 supplied by Thermo Fischer (Waltham, MA, USA). Samples were diluted 1:10 with 5 *v*/*v*% nitric acid and calibration standards of concentrations 0.1, 1.0 and 10.0 mg/L of the corresponding REE were prepared by diluting certified standard solutions with 5 *v*/*v*% nitric acid. The following wavelengths were used for the analysis of La, Ce, Nd, Gd, Dy and Y: 379.478 nm, 380.152 nm, 378.425 nm, 342.247 nm, 400.045 nm and 360.073 nm, respectively. All the wavelengths were aligned to the axial mode.

pH was measured using a Thermo Scientific Orion Star A211 pH-metre with an ORION 8302BNUMD Ross Ultra pH/ATC Triode electrode. Prior to each test, the pH metre was calibrated using a three-point calibration method with pH buffers 2, 4 and 7.

FT-IR spectra were obtained using a Spectrum Two spectrometer supplied by PerkinElmer (Wellesley, MA, USA) with a universal ATR sampling accessory and a volatile cup. The wavenumber range was 4000–400 cm^−1^, with a resolution of 4 cm^−1^ and a wavelength increment of 1 cm^−1^. The analyses were conducted using 32 scans per sample and the background spectrum was obtained by scanning the empty and clean top plate.

### 2.3. Experimental Procedure

#### 2.3.1. Evaluation of Third-Phase Formation in the HFRLM System

**Figure 2:**
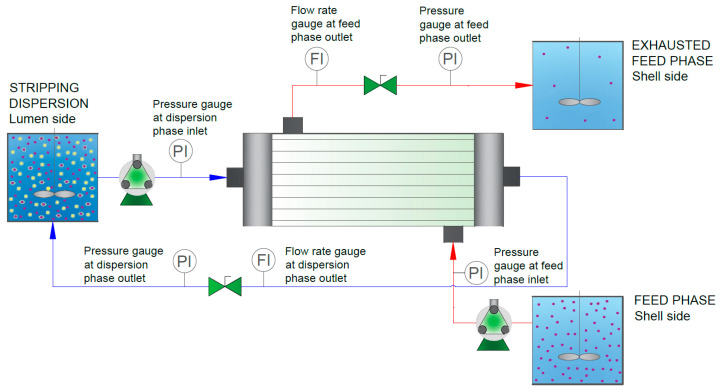
HFRLM setup.

The organic phase was prepared by diluting D2EHPA in kerosene in the absence or presence of a modifier. The stripping dispersion was prepared by mixing the stripping and the organic phases in an aqueous-to-organic phase volume ratio of A:O = 25. The volume of the stripping phase was 1.0 L, and the feed volume was 20 L. Prior to the experiment, the membrane fibres were impregnated with the organic phase for 60 min. Thereafter, the excess organic phase was removed from the system by pumping a small volume of water into both sides of the module. This step was performed at a low flow rate (40 to 50 mL/min) to avoid entraining the organic phase that impregnates the membrane pores by shear forces. After the impregnation step, the feed phase was pumped through the shell side of the fibres while the stripping dispersion was pumped through the lumen side (Figure 2). The interphase was maintained at the fibre pores by applying a higher pressure to the feed phase than to the dispersion phase. The differential pressure was always kept below the breakthrough pressure (typically between 0.2 and 0.4 bar [7,8,11]). The feed phase flowed continuously, and the dispersion was recirculated throughout the process. Both phases were circulated at a flow rate of 150 mL/min. Pressure manometers were employed as pressure gauges (PI), while rotameters were utilised as flow rate gauges (FI).

The molar flux of rare earth elements in the HFRLM process was evaluated using two distinct chemical systems (A and B), which differed in their feed and organic phase compositions. System A comprised a feed aqueous phase with a higher REE concentration (3.75 mM), higher feed pH (1.5–4.5) and lower D2EHPA concentration (10 *v*/*v*%) than system B, and the absence of a modifier. The composition of this system was expected to facilitate third-phase formation. System B involved a feed aqueous phase with the lowest REE concentration (1.55 mM), a low feed pH (0.6–2.5) and a slightly higher D2EHPA concentration (13 *v*/*v*%) than system A, and the addition of the modifier tri-n-butyl phosphate (1 *v*/*v*%). It was anticipated that this composition may have helped to prevent third-phase formation. The conditions tested in the HFRLM experiments are shown in Table 3. Both feed phases had the following molar composition: 16.0% La, 13.5% Ce, 15.4% Nd, 18.1% Gd, 17.0% Dy and 20.0% Y.

After each experiment, the membrane module was emptied and the stripping dispersion was recovered. During the HFRLM process, small aliquots of the exhausted feed phase and the stripping dispersion were taken at predetermined times. The experimental REE molar flux from the feed phase was determined as follows:

(3) $$J=\frac{Q_{f}\left({\left[RE\right]}_{f}^{in}-{\left[RE\right]}_{f}^{out}\right)}{A}$$

where J, Q_f_, A, ${\left[RE\right]}_{f}^{in}$ and ${\left[RE\right]}_{f}^{out}$ are the experimental molar flux (mol m^−2^ h^−1^), the flowrate in the feed phase (m^3^ h^−1^), the effective mass transfer area (m^2^) and the total concentrations of REE in the feed phase (mol m^−3^) at the inlet and the outlet of the membrane module, respectively.

The effective mass transfer area was calculated as follows:

(4) $$A=\varepsilon \times N\times 2\pi r_{0}l$$

where ε, N, r_0_ and l are the porosity, the number of fibres in the module, their outer radius (m) and their length (m), respectively.

#### 2.3.2. Determination of the Equilibrium Distribution Coefficient

To determine the equilibrium distribution of Y(III) during extraction and stripping, equal volumes of the aqueous and organic phases were stirred for 20 min, then allowed to settle and separate. For extraction tests, 5 mL of feed phase and 5 mL of fresh organic phase were used. For stripping tests, 4 mL of loaded organic phase and 4 mL of stripping phase were used. Y(III) solutions of exactly 41.1 mM at pH 1.5 and 2.5 were used as the feed phase, while 3M nitric acid solutions were used as the stripping phase. All experiments were carried out at room temperature (20.5 ± 0.5 °C).

The following concentrations of D2EHPA in kerosene were evaluated without the presence of a modifier: 10, 13, 16 and 20 *v*/*v*%. The effect of modifiers (tri-n-butyl phosphate or 1-decanol) on equilibrium was evaluated by adding 1, 3 and 5 *v*/*v*% modifiers to 10 *v*/*v*% D2EHPA diluted in kerosene.

The reaction between dimeric D2EHPA and REE ions is generally described by the following equilibrium [37,46,47]:

(5) $${RE}_{aq}^{3+}+{{3(HA)}_{2}}_{org}⇌{{{RE(HA}_{2})}_{3}}_{org}+{3H}_{aq}^{+}$$

where the subscripts aq and org denote aqueous and organic phases, respectively. RE^3+^ represents a rare earth ion and HA a protonated D2EHPA molecule.

The apparent equilibrium constant of extraction, K_eq_, is defined as follows:

(6) $$K_{eq}=\frac{\left[{RE{{(HA}_{2})}_{3}}_{org}\right]\cdot {\left[H_{aq}^{+}\right]}^{3}}{\left[{RE}_{aq}^{3+}\right]\cdot {\left[{{\left(HA\right)}_{2}}_{org}\right]}^{3}}=D\frac{{\left[H_{aq}^{+}\right]}^{3}}{{\left[{{\left(HA\right)}_{2}}_{org}\right]}^{3}}$$

and the distribution coefficient for REE extraction, D, is expressed as:

(7) $$D=\frac{\left[{RE{{(HA}_{2})}_{3}}_{org}\right]}{\left[{RE}_{aq}^{3+}\right]}=K_{eq}\frac{{\left[{{\left(HA\right)}_{2}}_{org}\right]}^{3}}{{\left[H_{aq}^{+}\right]}^{3}}$$

#### 2.3.3. Evaluation of the Limiting Organic Concentration (LOC)

The LOC is the maximum concentration of solute possible to load into the organic phase without causing third-phase formation. The LOC was determined by measuring the solute concentration in the aqueous phase at LOC conditions and by calculating the loaded solute in the organic phase by a mass balance.

All experiments were carried out at room temperature (20.5 ± 0.5 °C). Stock solutions of single Y(III) and mixed REEs were prepared in chloride media. The Y(III) solutions had a concentration of 40 ± 3.0 mM. The REE solution had a total REE concentration of 39.1 mM and contained 12.8% La, 10.6% Ce, 28.6% Nd, 14.1% Gd, 14.2% Dy and 19.7% Y. Unless otherwise stated, the organic phase used in the LOC tests was 10 *v*/*v*% D2EHPA in kerosene. The concentration of metals in the aqueous phase was determined by ICP-OES and the concentration in the organic phase was calculated by mass balance.

For the determination of the LOC, equal volumes (2 mL) of the aqueous feed and the organic phases were equilibrated in closed glass bottles and stirred for 20 min to reach equilibrium. The solution was allowed to settle and third-phase formation was determined visually by the appearance of turbidity and/or solid particles at the interphase. If no third phase was observed, 0.1 mL of the corresponding REE feed solution was added. The equilibration and separation procedures were performed again and repeated until third-phase formation. To this last solution, aliquots of fresh organic phase were gradually added until the third phase redissolved, i.e., until the organic phase became sufficiently transparent to allow a line to be clearly seen on the back side of the test vial. These were the LOC conditions at which the concentration in the aqueous phase was analysed, and the LOC in the organic phase was calculated as follows:

(8) $$LOC\left(\frac{mmol}{L}\right)=\frac{V_{f,LOC}\cdot {\left[RE\right]}_{f,0}-V_{f,LOC}\cdot {\left[RE\right]}_{f,LOC}}{V_{org,LOC}}=\frac{V_{f,LOC}\cdot \left({\left[RE\right]}_{f,0}-{\left[RE\right]}_{f,LOC}\right)}{V_{org,LOC}}$$

where V_f,LOC_ is the total volume of feed used for third-phase formation; [RE]_f,0_ is the initial concentration of solute in the feed phase; and [RE]_f,LOC_ and V_org,LOC_ are the concentrations of solute and the total organic volume at LOC conditions (i.e., when the third phase is redissolved), respectively. Approximately 15% of the LOC tests were randomly repeated to assess the repeatability of the LOC determination method. The coefficient of deviation for the repeated results was between 0.53 and 5.50%.

The loading of D2EHPA at LOC conditions, assuming all extractant molecules were in dimeric form and based on the reaction described in Equation (5), was calculated as:

(9) $$L\left(\%\right)=\frac{3*LOC}{\left[{\left(HA\right)}_{2_{org}}\right]}*100$$

The ratio of the volume of the aqueous feed phase to the volume of the organic phase at which the third-phase formation was detected in LOC experiments was calculated as:

(10) $${A:O}_{LOC}=\frac{V_{f,LOC}}{V_{org,LOC}}$$

The molar ratio between the D2EHPA and the REE amounts at the onset of third-phase formation was calculated as follows:

(11) $${D2EHPA:REE}_{LOC}=\frac{{\left[{\left(HA\right)}_{2}\right]}_{org}}{{\left[RE\right]}_{f,0}\cdot {A:O}_{LOC}}$$

The majority of experimental tests for LOC determination were performed with a single reference REE in order to facilitate the analysis of the results. The selection of this element was based on its hydration enthalpy, as it has been found to correlate with the interparticle potential energy of attraction. Previous studies have demonstrated that the nature of the extracted cation significantly influences third-phase formation, given that the size and charge of the cation affect the magnitude of the attractive intermicellar forces. Consequently, it is expected that cations with higher hydration energies will cause third-phase formation at lower concentrations in the organic phase, i.e., at lower LOCs [48,49]. Among the REEs in our mixture, the trend for the hydration enthalpy (−ΔH⁰_hyd_, kJ/mole) follows the following order: Y(3583) > Dy(3570) > Gd(3470) > Nd(3420) > Ce(3337) > La(3296) [50]. Accordingly, it was decided to select Y(III) as the reference element for LOC determination.

The impact of the feed pH on the LOC was examined within the pH range of 0.5 to 4.5 for the organic phase composed of 10 *v*/*v*% D2EHPA diluted in kerosene. At pH 0.5, the feed pH was adjusted by the addition of hydrochloric acid (Table 4a), whereas sodium hydroxide or ammonium hydroxide were used to adjust the pH of the feed solution between pH = 1.0 and 4.5 (Table 4b).

#### 2.3.4. Determination of Interactions in the Organic Phase: FT-IR Analysis

The molecular interactions between the extractant, the diluent and the modifier were investigated by FT-IR spectroscopy. Pure D2EHPA, TBP, 1-decanol and kerosene were analysed to identify the characteristic peaks of each substance. Diluted organic phases of D2EHPA in kerosene with and without modifiers were analysed. The effect of the equilibration of the organic phase for 10 min with distilled water and 40 mM Y(III) at pH 1.5 in an A:O ratio of 1 was also evaluated.

## 3. Results and Discussion

Key factors influencing the limiting organic concentration (LOC) and molar flux, such as feed pH, extractant concentration and the presence of modifiers, were systematically analysed to assess the conditions responsible for third-phase formation and its impact on the performance of hollow fibre liquid membrane systems.

### 3.1. HFRLM Evaluation

**Figure 3:**
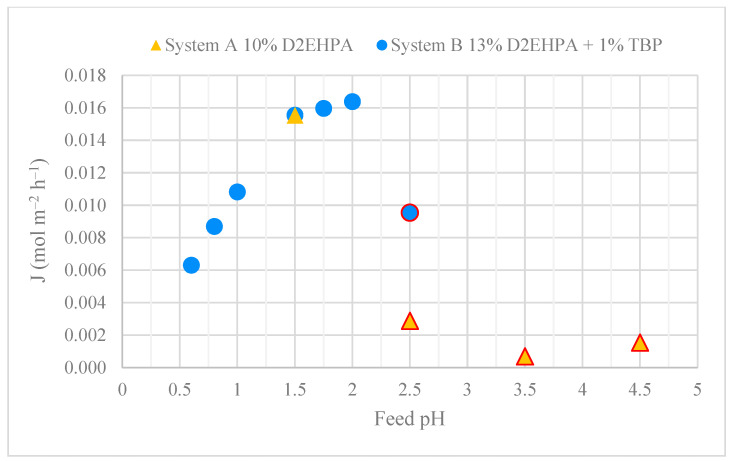
Molar flux in HFRLM for a mixture of REEs at different feed pH values. System A (yellow triangles): 3.75 mM mixed REEs as feed phase; 10 *v*/*v*% D2EHPA in kerosene as organic phase. System B (blue circles): 1.55 mM mixed REEs as feed phase; 13 *v*/*v*% D2EHPA + 1 *v*/*v*% TBP in kerosene as organic phase. Triangles and/or circles with a red line: visually detected third phase.

Both systems A and B exhibited comparable molar fluxes at an initial feed pH of 1.5, as shown in Figure 3. However, the molar flux for system A declined markedly at higher feed pH values (2.5–4.5), where a third phase was visually observed. The molar flux in system B increased significantly within the pH range of 0.6–1.5 and continued to rise more gradually between pH 1.5 and 2.0. It is suspected that third-phase formation began in the feed pH range of 1.75 to 2.0, as indicated by a slight decrease in the molar flux vs. feed pH. At pH 2.5, the third-phase formation was visually confirmed. A comparison of the performance of the two systems suggests that optimising the chemical composition can mitigate third-phase formation and enhance mass transfer, thereby improving HRFLM performance. Nevertheless, under the tested conditions, third-phase formation could not be entirely avoided in systems with a high pH and/or elevated REE concentration in the feed phase.

### 3.2. Determination of the Limiting Organic Concentration (LOC)

#### 3.2.1. Effect of Ions and pH in the Feed Aqueous Phase

The LOC is the highest concentration of solute that can be extracted into the organic phase without causing third-phase formation. A third phase forms when the organic phase becomes highly concentrated in REEs and aggregation or polymerisation phenomena are favoured. Therefore, the highest LOC values are achieved when experimental conditions promote REE extraction while preventing D2EHPA molecule aggregation. The determined LOC values and organic loading at LOC conditions at different feed compositions are presented in Table 4.

The results in Table 4a reveal two distinct LOC regions for the Y(III) solution: LOC values of approximately 9 mM at pH 0.5–2.0 and approximately 4 mM at pH 2.0–4.5. The organic phase loading at the LOC was estimated to be 17–20% for the first region and 7–9% for the second. The change in LOC values around pH 2.5 is likely attributed to the increase in D2EHPA dissociation (pKa = 1.47–2.79 [51,52,53]). While D2EHPA solubility increases with rising pH, this effect is negligible at an aqueous phase pH below 4.0 [54]. Therefore, LOC values are higher at lower pH because REE extraction efficiency decreases when D2EHPA is either undissociated or only partially dissociated. Furthermore, D2EHPA aggregation becomes more pronounced at high pH levels provided the acid concentration is not high enough for acid extraction by D2EHPA [55].

The use of ammonia for pH adjustment resulted in slightly lower LOC values compared to sodium hydroxide. Although the hydration enthalpy of sodium is higher than that of the ammonium cation [50], the difference has a negligible effect on third-phase formation when these salts are used at low concentrations. Based on these similar results, sodium hydroxide (NaOH) was selected for pH adjustment in the subsequent sections.

**Figure 4:**
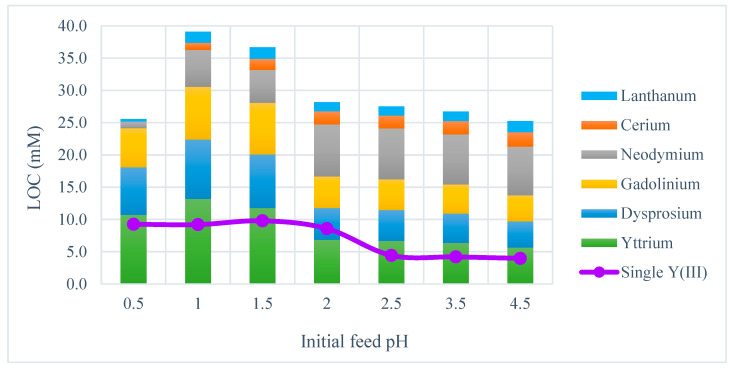
Effect of pH in the initial feed on the LOC of a multicomponent REE solution (bar) vs. the LOC for a single Y(III) solution (line).

Furthermore, the effect of feed pH on the LOC was evaluated for a REE mixture with an equivalent total molar concentration to the yttrium solution. The LOC values and the loading in the organic phase at the LOC for the REE mixture were considerably higher than those for the single Y(III) solution over the whole range of investigated pH (Table 4b and Figure 4). It is particularly noteworthy that the yttrium concentration in the organic solution at LOC conditions for the REE mixture was generally higher than at the LOC for the single yttrium solution. This supports the hypothesis that yttrium is the REE most prone to promoting third-phase formation. This also suggests that the presence of other REEs less prone to third-phase formation may have a preventive effect. The LOC values for the REE mixture were significantly higher at feed pH 1.0 and 1.5 and lower with a slight decrease over the pH range 2.0–4.5. This is consistent with the observations in the HFRLM extraction experiments, where a decreased flux was measured in the higher feed pH range at and above 1.75 and there was a visual observation of third-phase formation and significantly decreased flux at pH 2.5 and above, as seen in Figure 3. The LOC at pH 0.5 was significantly lower than at pH 1.0 and 1.5, likely due to the low concentrations of light REEs (La, Ce and Nd) in the organic phase. This results from the high selectivity of D2EHPA for heavy REEs at a low feed pH [56]. The increasing concentrations of La, Ce and Nd in the organic phase at pH 1.0 and 1.5 significantly raised the LOC, although the feed pH became the dominant factor from pH 2.0 onwards.

**Figure 5:**
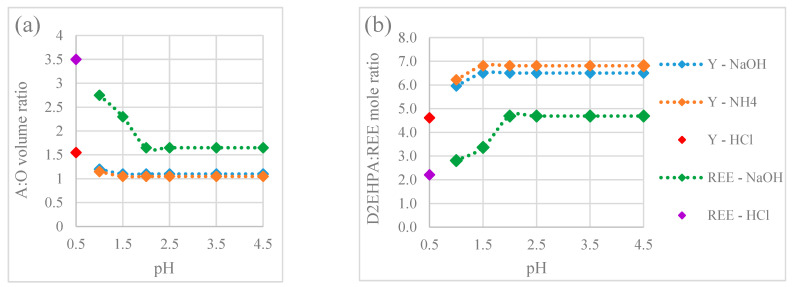
Conditions leading to the formation of the third phase: (**a**) Aqueous to organic (A:O) volumetric ratio; (**b**) D2EHPA to yttrium or mixed REEs molar ratio. Feed pH at pH 0.5 is adjusted with HCl and the feed pH between 1.0 and 4.5 is adjusted with NaOH or NH_4_ solutions.

The onset of the third-phase formation at different A:O ratios and D2EHPA:yttrium molar ratios are shown in Figure 5. The third phase was formed at significantly higher A:O ratios and lower D2EHPA:REE molar ratios for the REE mixture than for the yttrium solution, particularly within the pH range of 0.5–1.5. These findings indicate that extracting a mixture of light and heavy REEs at a low feed pH (0.5–1.5) may lead to less third-phase formation compared to extracting only yttrium or extracting the mixed REEs at a higher feed pH (2.0–4.5). Furthermore, the significantly lower minimum D2EHPA:metal molar ratio at the onset of the third-phase formation for the REE mixture at a lower feed pH underscores the importance of selecting the appropriate aqueous phase in systems with limited volumes of organic phase.

#### 3.2.2. Effect of Organic Diluent

**Figure 6:**
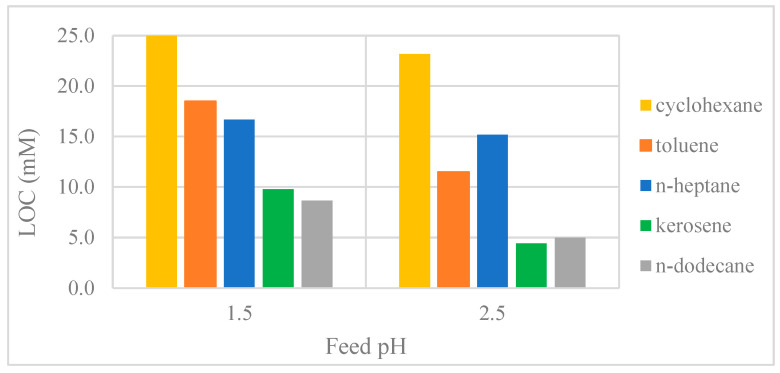
Effect of organic diluent on LOC.

As shown in Figure 6, the use of various organic diluents reduced the LOC and maximised organic loading in the following order: cyclohexane > toluene ≈ n-heptane > kerosene ≈ n-dodecane.

The relatively high LOC in the toluene system can be attributed to the stronger interaction between aromatic diluents and organophosphorus acid extractants, compared to aliphatic diluents. Consequently, the formation of hydrogen bonds between the D2EHPA monomers and the aromatic diluent competes with the formation of hydrogen bonds between the D2EHPA monomers, reducing the aggregation of D2EHPA in the organic phase [57]. The increased interaction between D2EHPA and the aromatic diluent leads to a significant reduction in both the equilibrium constants and extraction efficiency of REEs [35].

Diluents with shorter and more branched chains exhibit a higher degree of penetration, which swells the apolar layer of the reverse micelles. A thicker apolar layer reduces the attractive van der Waals forces between the polar cores of the micelles, thereby increasing the stability of the organic phase [31]. Therefore, the use of short and/or branched aliphatic diluents (cyclohexane, n-heptane) is more effective in preventing the third-phase formation than the use of diluents containing long aliphatic chains (n-dodecane).

Kerosene was selected for the majority of the experiments in this study, as it contains both aliphatic and aromatic compounds, which provide high extraction performance and a relatively stable organic phase. This, in combination with good availability and price, makes it a suitable diluent in industrial processes for REE separation.

#### 3.2.3. Effect of Extractant Concentration

**Figure 7:**
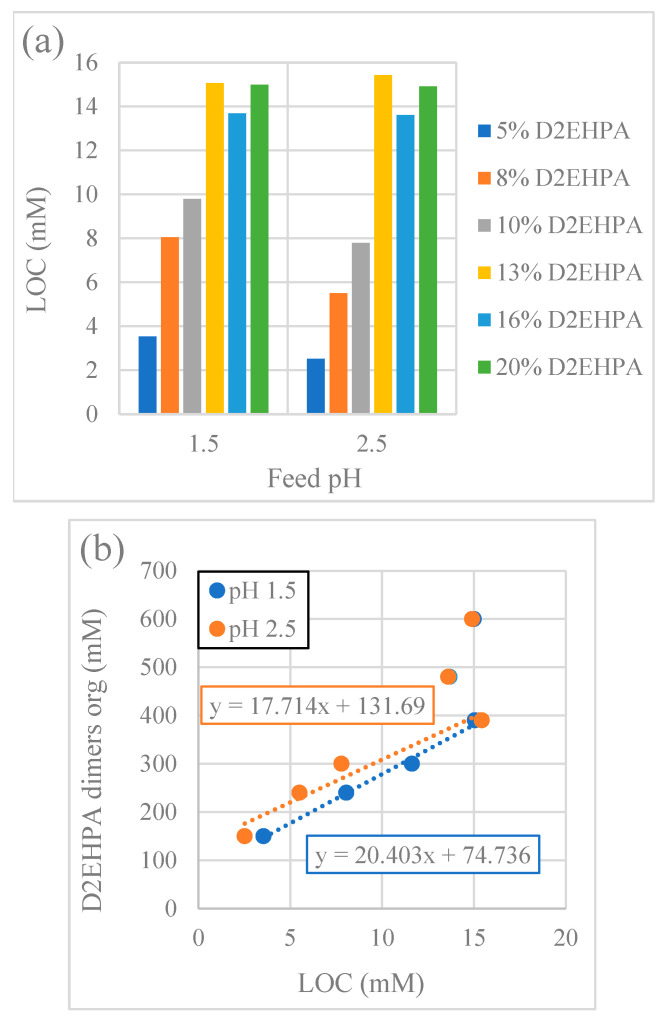
(**a**) Effect of D2EHPA concentration on LOC in Y(III) solution; (**b**) linear relationship between D2EHPA concentration (mM) and LOC in Y(III) solution (mM).

The LOC values for various extractant concentrations were generally lower at feed pH 2.5 than at feed pH 1.5 (Figure 7a), in accordance with the results presented in Table 4 for a D2EHPA concentration of 10 *v*/*v*% in kerosene. The LOC increased linearly with a D2EHPA concentration between 5 and 13 *v*/*v*% D2EHPA (equivalent to 150–390 mM dimeric D2EHPA) at both feed pH levels (Figure 7b). However, this linear trend did not continue for the 16–20 *v*/*v*% D2EHPA concentration range. These results suggest that either the third-phase formation occurs at lower D2EHPA loadings, or alternatively, that the stoichiometry of the REE-D2EHPA complexes in the third phase changes at higher D2EHPA concentrations. A change in stoichiometry would be consistent with the observations of Mohammadi et al. (2015) [51], which identified a possible change in the stoichiometry of the REE-D2EHPA complexes at higher loading. The change in stoichiometry was indicated by a relatively higher increase in the percentage of D2EHPA associated with REEs at lower D2EHPA concentrations as the equilibrium pH increased in the investigated pH range of 0.8 to 1.5. The equilibrium tests indicated a tendency towards an increased extraction efficiency and distribution ratio at a higher D2EHPA concentration and feed pH.

The concentration of D2EHPA significantly affects the onset of third-phase formation. As illustrated in Table 5, the third phase was observed at much higher A:O ratios with increased extractant concentrations. This indicates that higher concentrations of D2EHPA in the organic phase prevent the formation of the third phase. Furthermore, the molar ratio between the available D2EHPA and Y(III) at the onset of the third-phase formation, within the D2EHPA concentration range of 10 to 20 *v*/*v*%, was between 5.2 and 6.5. The feed pH had a negligible effect on both ratios at the investigated pH values of 1.5 and 2.5.

It is challenging to determine the A:O ratio and the D2EHPA:REE mole ratio in the HFRLM extraction experiments. However, the results support the observation of lower flux in the experiment at a feed pH of 2.5 in system A (higher REE concentration) with 10% D2EHPA compared to system B (lower REE concentration) with 13% D2EHPA, as illustrated in Figure 3. It should be noted that, in contrast to system B, system A contained a phase modifier, which also had an effect on the results.

#### 3.2.4. The Effect of Adding a Modifier

As both the extractant and the metal complexes are polar, they exhibit high phase incompatibility and a low interaction with apolar aliphatic diluents. To increase their solubilisation in the organic phase, a modifier can be incorporated [57]. Modifiers are typically highly hydrophobic and amphiphilic molecules that compete with the extractant for adsorption to the micelle surface [58].

##### The Effect of Tri-N-Butyl Phosphate Addition

The addition of tri-n-butyl phosphate (TBP) increased the LOC, the maximum loading and the A:O ratio at the onset of third-phase formation under all conditions tested. The highest LOC and loading values were observed for TBP concentrations of 1, 4 and 5% *v*/*v* while the highest A:O ratios at the onset of third-phase formation corresponded to the addition of 1–3 *v*/*v*% TBP (Table 6). The addition of TBP to the organic phase reduced the impact of pH on LOC in the pH range 1.5–2.5. The results support the findings from the HFRLM extraction experiments at a feed pH of 2.5, where system A containing a phase modifier and a higher D2EHPA concentration performed better in terms of flux compared to system B, as seen in Figure 3.

The addition of TBP in the organic phase increased the yttrium extraction only at a low TBP concentration. The extraction efficiency was high in the presence of 1 and 3 *v*/*v*% of TBP, while the stripping efficiency was high with the presence of 0 and 5 *v*/*v*% TBP (Table 7). It should be noted that the extraction efficiency increased with the distribution ratio (D_extraction_), while the stripping efficiency increased as the distribution ratio (D_stripping_) decreased. The recovery efficiency, which considered both the extraction and stripping stages, followed the order 0 > 5 > 3 > 1 *v*/*v*% TBP.

The FT-IR spectra showed that the addition of TBP to the organic phase caused a shift in D2EHPA’s phosphoryl band (P=O, 1225 cm^−1^) to higher wavelengths, indicating the partial conversion of D2EHPA dimers to the monomeric form (see SI for FT-IR data). Furthermore, the characteristic bands of both extractants were detected in the spectra, confirming that the influence of TBP on the distribution ratios of yttrium was due to its role as a phase modifier, rather than a synergistic effect between D2EHPA and TBP. The presence of bulk water, detectable by an increase in the band at 3400 cm^−1^, was not observed in the organic phase when equilibrated with water or an yttrium solution (see SI for FT-IR data).

##### The Effect of the Addition of 1-Decanol

As shown in Table 8, the addition of 1 *v*/*v*% 1-decanol reduced the LOC and the maximum D2EHPA loading. The presence of 4–6 *v*/*v*% 1-decanol decreased the A:O volume ratio at the onset of the third-phase formation, while 1–3% *v*/*v* 1-decanol slightly increased the volume ratio.

The addition of 1-decanol slightly reduced the extraction efficiency, with this effect becoming more pronounced as the concentration of 1-decanol increased. However, stripping efficiency—and thus the overall recovery efficiency—improved with the addition of 1-decanol. Therefore, both the distribution ratios for the extraction and for the stripping of Y(III) decreased as the concentration of 1-decanol in the organic phase increased at both feed pH values (Table 9). The extraction efficiency followed the following order: 0 > 1 > 3 > 5 *v*/*v*% of 1-decanol, while the stripping efficiency followed the order of 0 > 5 > 3 > 1 *v*/*v*% of 1-decanol.

The FT-IR spectra showed that the addition of 1-decanol led to a shift in the band of the phosphoryl bond to higher wavelengths, indicating the partial conversion of D2EHPA dimers to the monomeric form. No significant changes were observed in other characteristic bands for D2EHPA (P-O-C/P-O-H, 1012 cm^−1^) and the presence of bulk water (3400 cm^−1^) was not detected (see SI for FT-IR data).

The mechanism by which 1-decanol contributes to the third-phase formation and affects the extraction and stripping processes is therefore not yet fully understood. It is known that long-chain alcohols can act as cosurfactants, competing with D2EHPA to achieve adsorption at the aqueous–organic interface [59]. This competition may reduce the availability of D2EHPA molecules at the interface, which is thought to be the mechanism by which 1-decanol promotes the third-phase formation and decreases extraction efficiency.

## 4. Conclusions

This study explored the factors that influence third-phase formation during the extraction of rare earth elements (REEs) using D2EHPA in hollow fibre renewal liquid membrane (HFRLM) systems. Third-phase formation is a key issue that reduces mass transfer and causes membrane fouling, leading to lower efficiency in REE extraction processes. Therefore, understanding the factors that promote or prevent third-phase formation is crucial for improving HFRLM performance.

It was found that yttrium is more prone to form a third phase compared to a mixture of light and heavy REEs. The presence of other REEs in the mixture helped to increase the limiting organic concentration (LOC) and delayed third-phase formation. This study also showed that higher feed pH values, especially above 2.0, promoted third-phase formation. At a low pH, the third phase was less likely to form due to the reduced aggregation of D2EHPA. The organic phase composition also played an important role. Low D2EHPA concentrations (5–10 *v*/*v*%) promoted third-phase formation, while higher D2EHPA concentrations increased the LOC and improved phase stability. The type of diluent was significant as well. Linear aliphatic diluents caused more third-phase formation, while cyclic and aromatic diluents, like cyclohexane, were more effective at preventing it. Kerosene, a mixture of aliphatic and aromatic compounds, was found to be the most practical diluent due to its balance of extraction performance and stability.

Adding a modifier such as tri-n-butyl phosphate (TBP) improved phase stability and increased the LOC, preventing third-phase formation under all tested conditions. In contrast, 1-decanol lowered LOC and increased the likelihood of third-phase formation, especially at higher concentrations. This shows that the choice of modifier has a significant impact on system stability.

The HFRLM experiments confirmed that the feed and organic phase composition strongly influence third-phase formation and mass transfer. Using 13 *v*/*v*% D2EHPA in kerosene with 1 *v*/*v*% TBP prevented third-phase formation when the feed pH was between 0.6 and 2.0, but third-phase formation occurred at pH 2.5. Higher REE concentrations and feed pH made it harder to prevent third-phase formation, especially with heavy REEs present.

In conclusion, this study identified the key factors affecting third-phase formation during REE extraction with D2EHPA. Feed pH, the type of REE, the concentration of extractant and the choice of diluent and modifiers all play a role in determining phase stability. This research provides useful insights for optimising HFRLM systems to prevent third-phase formation and improve efficiency in industrial processes.

## Figures and Tables

**Table 1:** Summary of previous studies on metal extraction by means of HFRLMs.

Ref.	Author	Year	Feed Phase	Organic Phase	Stripping Phase
[9]	K. Hu et al.	2023	Y(III)	[N1888] [CA12] in kerosene	HCl/Deionised water
[10]	M. Alemrajabi et al.	2022	Mixed REEs (La, Nd, Sm, Dy, Er, Y) in chloride media	D2EHPA in kerosene	HCl
[11]	A. Yadollahi et al.	2019	Zr/Hf in nitrate media	Mixture of TBP and Cyanex 272	NH_4_F
[12]	F. Zahakifar et al.	2018	U(VI) in sulphate media	Alamine 336 in kerosene	NaCl, NH_4_Cl, NaHCO_3_
[13]	F. Zahakifar et al.	2018	U(VI) in sulphate media	Alamine 336 in kerosene	NaCl, NH_4_Cl, NaNO_3_, NaF, Na_2_CO_3_, NaHCO_3_
[14]	Cheng H. et al.	2017	Yb(III) in chloride media	EHEHPA in n-heptane	HCl
[8]	S.A. Allahyari et al.	2017	Th(IV) in nitrate media	Cyanex 272 in kerosene	H_2_SO_4_
[15]	S.A. Allahyari et al.	2016	Th(IV) in nitrate media	TBP in kerosene	HNO_3_
[16]	Z. Ren et al.	2013	CuSO_4_	LIX984N in kerosene	H_2_SO_4_
[17]	l. Pei et al.	2012	Sm(III) in acetate-buffered media	PC-88A/EHEHPA in kerosene	HNO_3_
[18]	L. Pei et al.	2011	Sm(III) in acetate-buffered chloride media	P204/D2EHPA in kerosene	HCl
[19]	L. Pei et al.	2011	Dy(III) in acetate-buffered media	PC-88A/EHEHPA in kerosene	HCl
[20]	Z. Ren et al.	2010	Cu(II) and Co(II) in sulphate media	LIX984N in kerosene for Cu(II)/Cyanex 272 in kerosene for Co(II)	H_2_SO_4_
[21]	W. Zhang	2010	CuSO_4_ in acetate buffer solution	D2EHPA or LIX984N in kerosene	H_2_SO_4_
[22]	W. Zhang	2010	CuSO_4_ in sulphate media	LIX984N in kerosene	H_2_SO_4_
[23]	L. Pei et al.	2009	Tm in acetate-buffered chloride media	PC-88A/EHEHPA in kerosene, n-heptane, methyl-isobutyl, ketone, butylacetate and benzene	HCl
[24]	Z. Ren et al.	2008	CuSO_4_ in acetate buffer solution	D2EHPA in kerosene	HCl, H_3_PO_4_, H_2_SO_4_, HNO_3_
[7]	Z. Ren et al.	2007	CuSO_4_	D2EHPA in kerosene	HCl

**Table 2:** Hollow fibre contactor characteristics according to specifications from the provider.

Provider	3M
Contactor model	Liqui-Cel Extra-Flow 2.5 in × 8 in Celgard X50-215 Microporous Hollow Fibre Membrane Polypropylene Fibre
Effective surface area (m^2^)	1.4
Effective fibre length (cm)	20.32
Number of fibres	11,000
Outer fibre diameter (μm)	300
Inner fibre diameter (μm)	220
Fibre wall thickness (μm)	40
Fibre porosity	0.4
Pore tortuosity	2.5
Effective pore diameter (μm)	0.04

**Table 3:** Experimental conditions for HFRLM tests.

	Exp.	[REE]_feed_ (mM)	pH_feed_	[D2EHPA]_org_ (*v*/*v*%)	[TBP]_org_ (*v*/*v*%)	[HNO_3_]_strip_ (M)
A	1	3.75	1.5	10%	-	3
	2	3.75	2.5	10%	-	3
	3	3.75	3.5	10%	-	3
	4	3.75	4.5	10%	-	3
B	5	1.55	0.6	13%	1%	3
	6	1.55	0.8	13%	1%	3
	7	1.55	1.0	13%	1%	3
	8	1.55	1.5	13%	1%	3
	9	1.55	1.75	13%	1%	3
	10	1.55	2.0	13%	1%	3
	11	1.55	2.5	13%	1%	3

**Table 4:** LOC (mM) and organic loading at LOC conditions (L, %) in 10 *v*/*v*% D2EHPA in kerosene. Feed aqueous phase at pH ranging from 0.5 to 4.5 and compositions of (**a**) 42.3 mM Y(III) and (**b**) 39.1 mM mixed REE(III).

	(a) Y(III) Solution						(b) REE Mixture		
Feed pH	pH Adjuster	LOC (mM)	L (%)	pH Adjuster	LOC (mM)	L (%)	pH Adjuster	LOC (mM)	L (%)
0.5	HCl	9.3	18.4	-	-	-	HCl	25.6	50.7
1.0	NaOH	9.2	18.3	NH_4_OH	9.5	18.9	NaOH	39.1	77.6
1.5	NaOH	9.8	19.4	NH_4_OH	9.2	18.3	NaOH	36.7	71.7
2.0	NaOH	8.6	17.0	NH_4_OH	4.5	9.0	NaOH	28.2	55.9
2.5	NaOH	4.4	8.8	NH_4_OH	4.3	8.5	NaOH	27.5	54.6
3.5	NaOH	4.2	8.3	NH_4_OH	3.5	6.9	NaOH	26.7	53.0
4.5	NaOH	4.0	7.9	NH_4_OH	3.4	6.8	NaOH	25.3	50.1

**Table 5:** Aqueous-to-organic volume ratio (A:O, mL/mL) and D2EHPA:Y(III) ratio (mol/mol) at third-phase onset.

	Feed pH		
	1.5–2.5	1.5	2.5
Organic Phase	A:O Ratio	D2EHPA:Y Ratio	
5 *v*/*v*% D2EHPA	<1	-	-
8 *v*/*v*% D2EHPA	<1	-	-
10 *v*/*v*% D2EHPA	1.15	6.0	6.0
13 *v*/*v*% D2EHPA	1.7	5.2	5.2
16 *v*/*v*% D2EHPA	1.7	6.4	6.5
20 *v*/*v*% D2EHPA	2.2	6.2	6.2

**Table 6:** The effect of tri-n-butyl phosphate addition to 10 *v*/*v*% D2EHPA diluted in kerosene on the LOC. The effect_LOC_ (%) is expressed as a percentage variation from the LOC values obtained in the absence of a modifier. Negative effect values represent a decrease while positive values represent an increase.

		pH 1.5			pH 2.5			pH 1.5–2.5
[TBP] (*v*/*v*%)	[TBP] (M)	LOC (mM)	Effect_LOC_ (%)	L (%)	LOC (mM)	Effect_LOC_ (%)	L (%)	A:O Ratio
1	0.04	13.8	+90.3	27.44	13.8	+76.7	27.28	1.25
2	0.07	10.5	+45.0	20.91	11.6	+48.7	22.95	1.25
3	0.11	9.2	+26.8	18.28	9.2	+17.7	18.17	1.25
4	0.15	14.5	+99.9	28.83	14.5	+85.7	28.67	1.15
5	0.18	14.5	+100.0	28.85	14.5	+85.7	28.67	1.15
6	0.22	11.3	+55.5	22.43	11.2	+44.3	22.29	1.15

**Table 7:** Distribution ratios for the extraction and stripping of yttrium(III) in 10 *v*/*v*% D2EHPA diluted in kerosene at various TBP concentrations (phase modifier).

Feed Phase		Organic Phase		
pH	[Y]_feed_ (mM)	[TBP] (*v*/*v*%)	D_Extraction_	D_Stripping_
1.5	41.11	0	91.1	0.27
		1	318.9	0.46
		3	130.2	0.37
		5	51.6	0.32
2.5	41.05	0	96.3	0.31
		1	546.9	0.45
		3	175.6	0.41
		5	106.74	0.34

**Table 8:** Effect of the addition of 1-decanol to 10 *v*/*v*% D2EHPA diluted in kerosene on the LOC. The effect_LOC_ (%) is expressed as a percentage variation from the LOC values obtained in the absence of a modifier. Negative effect values represent a decrease while positive values represent an increase. The concentration of 1-decanol is expressed with the term [1-dec].

		pH 1.5			pH 2.5			pH 1.5–2.5
[1-Dec] (*v*/*v*%)	[1-Dec] (M)	LOC (mM)	Effect_LOC_ (%)	L (%)	LOC (mM)	Effect_LOC_ (%)	L (%)	A:O Ratio
1	0.05	4.8	−33.8	9.55	5.2	−32.7	10.39	1.25
2	0.10	5.7	−22.2	11.22	4.6	−40.9	9.13	1.15
3	0.15	5.7	−22.2	11.22	6.8	−13.3	13.39	1.15
4	0.21	6.3	−13.0	12.54	6.3	−19.2	12.47	1.0
5	0.26	5.9	−18.9	11.70	5.9	−24.6	11.64	1.0
6	0.31	6.3	−13.1	12.53	5.5	−29.4	10.91	1.0

**Table 9:** Distribution ratios for the extraction and stripping of yttrium (III) in 10 *v*/*v*% D2EHPA diluted in kerosene with different concentrations of 1-dec (1-decanol).

Feed Phase		Organic Phase		
pH	[Y]_feed_ (mM)	[1-Dec] (*v*/*v*%)	D_extraction_	D_stripping_
1.5	41.11	0	91.1	0.27
		1	17.2	0.18
		3	14.4	0.13
		5	9.3	0.11
2.5	41.05	0	96.3	0.31
		1	29.7	0.19
		3	26.3	0.16
		5	17.9	0.11

## Data Availability

The original contributions presented in this study are included in the article/Supplementary Materials. Further inquiries can be directed to the corresponding author(s).

## Supplementary Materials

The following supporting information can be downloaded at: https://www.mdpi.com/article/10.3390/membranes15070188/s1, Figure S1: FT-IR spectra for 10% *v*/*v* D2EHPA diluted in kerosene. Blue: no contact with aqueous phase; orange: organic phase equilibrated with distilled water; green: organic phase loaded with Y(III); fuchsia: third phase loaded with Y(III); Figure S2: FT-IR spectra of 10 *v*/*v*% D2EHPA with 5 *v*/*v*% TBP and 1-decanol; Figure S3: FT-IR spectra of 10 *v*/*v*% D2EHPA in kerosene with 1, 3, and 5 *v*/*v*% TBP equilibrated with an yttrium solution and a third phase formed in 10 *v*/*v*% D2EHPA with 1 *v*/*v*% TBP in kerosene; Figure S4: FT-IR spectra of 10 *v*/*v*% D2EHPA in kerosene with 1, 3, and 5 *v*/*v*% 1-decanol equilibrated with an yttrium solution and a third phase formed in 10 *v*/*v*% D2EHPA with 1 *v*/*v*% 1-decanol in kerosene. Table S1: Characteristic FT-IR peaks of individual components of the organic phase.
