# Supplementary material for: Third-Phase Formation in Rare Earth Element Extraction with D2EHPA: Key Factors and Impact on Liquid Membrane Extraction Performance
Source: Membranes (Basel). 2025 Jun 23;15(7):188. doi: 10.3390/membranes15070188 (PMC12300411; doi:10.3390/membranes15070188)
Supplement: Supplementary file 1 [file membranes-15-00188-s001.zip › membranes-3678821-supplementary.pdf]

SUPPORTING INFORMATION

FT-IR: Interactions in the organic phase

The FT-IR spectra of individual components and of the extractant diluted in organic diluent with and without modifiers were analysed. The effect of the equilibration of the organic phase with distilled water and an yttrium-rich solution was also evaluated. The characteristic FT-IR peaks of the different components of the organic phase are listed in Table S1.

Table S1. Characteristic FT-IR peaks of individual components of the organic phase.

D2HEPA ($C_{16}H_{35}O_4P$)		
P=O	1225.31 cm^{-1}	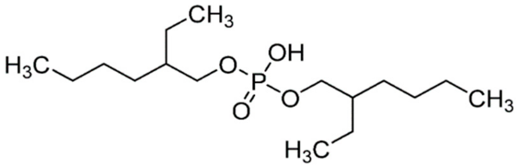
P-O-C/P-O-H	1012.25 cm^{-1}	
CH_x stretching vibrations	2958.77 cm^{-1}<br>2929.97 cm^{-1}<br>2873.83 cm^{-1}<br>2860.72 cm^{-1}	
CH_x bending vibrations	1462.43 cm^{-1}<br>1380.78 cm^{-1}	
Kerosene (C_nH_{2n+2}, C=10-16)		
C-H asymmetric stretching	2954.40 cm^{-1}<br>2921.90 cm^{-1}	
C-H symmetric stretching	2853.71 cm^{-1}	
CH_2 bending vibrations	1458.20 cm^{-1}<br>1377.40 cm^{-1}	
TBP ($C_{12}H_{27}O_4P$)		
P=O	1279.86 cm^{-1}	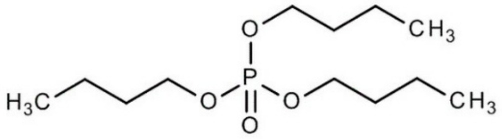
P=O stretching vibrations	1265.10 cm^{-1}<br>1235.83 cm^{-1}	
CH_x stretching vibrations	2959.71 cm^{-1}<br>2935.44 cm^{-1}<br>2903.37 cm^{-1}<br>2874.76 cm^{-1}	
CH_x bending vibrations	1465.11 cm^{-1}<br>1433.36 cm^{-1}<br>1382.17 cm^{-1}	

C–O–(P) out of phase	1058.82 cm^{-1}	
C–O–(P) in phase	1020.55 cm^{-1}	
P–O–(C)	977.42 cm^{-1}	
P–O–(C) stretch/CH_3 wagging	908.79 cm^{-1}	
P-O_3 symmetric stretch	735.46 cm^{-1}	
1-decanol ($C_{10}H_{22}O$)		
O-H stretching vibration	3325.14 cm^{-1}	
CH_x stretching vibration	2956.28 cm^{-1}<br>2922.59 cm^{-1}<br>2853.65 cm^{-1}	
CH_2 scissoring	1465.54 cm^{-1}	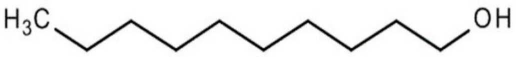
CH_2 twisting	1252.78 cm^{-1}	
CH_2 rocking	721.06 cm^{-1}	
C-C stretching vibration / OH bending vibration	1056.75 cm^{-1}	

A.1. D2EHPA–kerosene system

The equilibration of 10% D2EHPA in kerosene with water had a negligible effect on the organic phase. However, the equilibration with an yttrium-rich solution caused a significant shift in the phosphoryl band (from 1230.22 cm^{-1} to 1204.36 cm^{-1}), corresponding to the Y-D2EHPA complex. The P-O-H/P-O-C peak exhibited a slight shift (from 1033.27 cm^{-1} to 1031.53 cm^{-1}) and a reduction in absorbance intensity, indicative of the release of hydrogen from the P-O-H bond due to metal extraction (Fig. S1). Comparable behaviour was observed in the corresponding third phase, where the phosphoryl band was located at 1202.87 cm^{-1} and the P-O-H/P-O-C peak was observed at 1302.03 cm^{-1}.

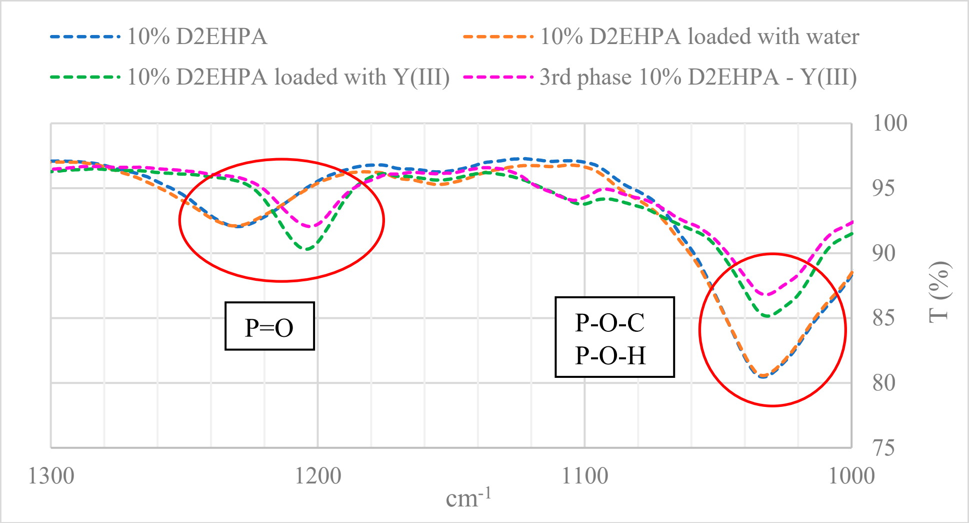

Figure S1. FT-IR spectra for 10% v/v D2EHPA diluted in kerosene. Blue: no contact with aqueous phase; orange: organic phase equilibrated with distilled water; green: organic phase loaded with Y(III); fuchsia: third phase loaded with Y(III).

A.2. D2EHPA–modifier–kerosene systems

As illustrated in Figure S2, the addition of either TBP or 1-decanol in a concentration range from 1 to 5 v/v% to 10 v/v% D2EHPA diluted in kerosene shifted the phosphoryl band to higher wavelengths (1% TBP: 1230.56 cm^{-1}; 3% TBP: 1235.14 cm^{-1}; 5% TBP: 1249.18 cm^{-1}; 1% 1-decanol: 1234.72 cm^{-1}; 3% 1-decanol: 1236.44 cm^{-1}; 5% 1-decanol: 1237.46 cm^{-1}), indicating the weakening of hydrogen bonds in D2EHPA and the conversion of some dimers to a monomeric form. The addition of TBP shifted the P-O-C/P-O-H band to lower wavelengths and increased its intensity (1% TBP: 1031.49 cm^{-1}, 0.09 A; 3% TBP: 1029. 84 cm^{-1}, 0.12 A; 5% TBP: 1028.80 cm^{-1}, 0.13 A), which can be attributed to the overlap of TBP's P-O-(C) band with D2EHPA´s. The addition of 1-decanol had a negligible effect on the P-O-C/P-O-H band.

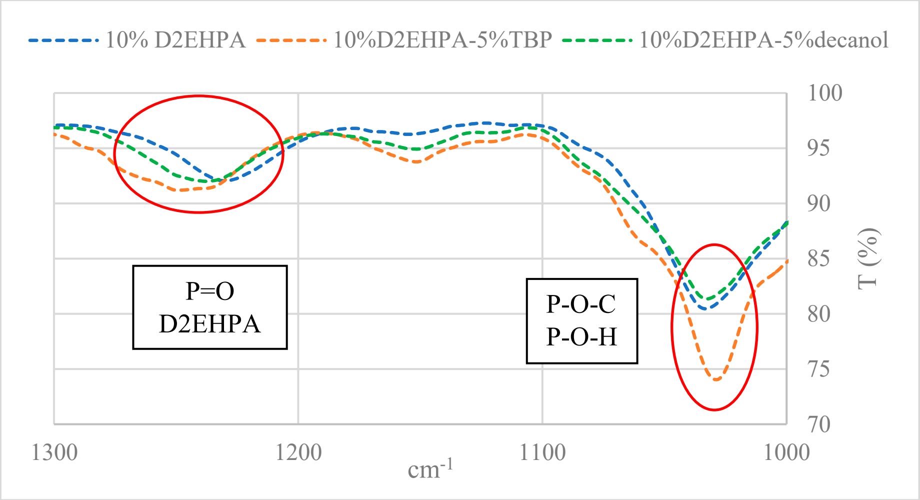

Figure S2. FT-IR spectra of 10 v/v % D2EHPA with 5 v/v % TBP and 1-decanol.

Y(III) loading of the organic phase shifted the P=O D2EHPA band to lower wavelengths (1% TBP: 1204.21 cm^{-1}; 3% TBP: 1204.06 cm^{-1}; 5% TBP: 1204.16 cm^{-1}; 3rd phase 1% TBP: 1999.99 cm^{-1}) due to coordination with the metal. The P-O-C/P-O-H band shifted to slightly lower

wavelengths and its intensity decreased (1% TBP: 1030.27 cm^{-1}, 0.07 A; 3% TBP: 1028.84 cm^{-1}, 0.09 A; 5% TBP: 1028.21 cm^{-1}, 0.11 A; 3rd phase 1% TBP: 1030.86 cm^{-1}, 0.07 A), indicating the release of H in the P-O-C band due to complexation. Furthermore, at higher TBP concentrations, the P=O band corresponding to TBP was detected (3% TBP: 1271. 42 cm^{-1}, 0.02 A; 5% TBP: 1271.46 cm^{-1}, 0.03 A). This suggests that the P=O band for TBP was masked by D2EHPA's due to its low concentration in the organic phase. The fact that the characteristic bands of both extractants were detected in the spectrum indicates that there is no interaction between the extractants, i.e., no synergistic effect (Fig. S3).

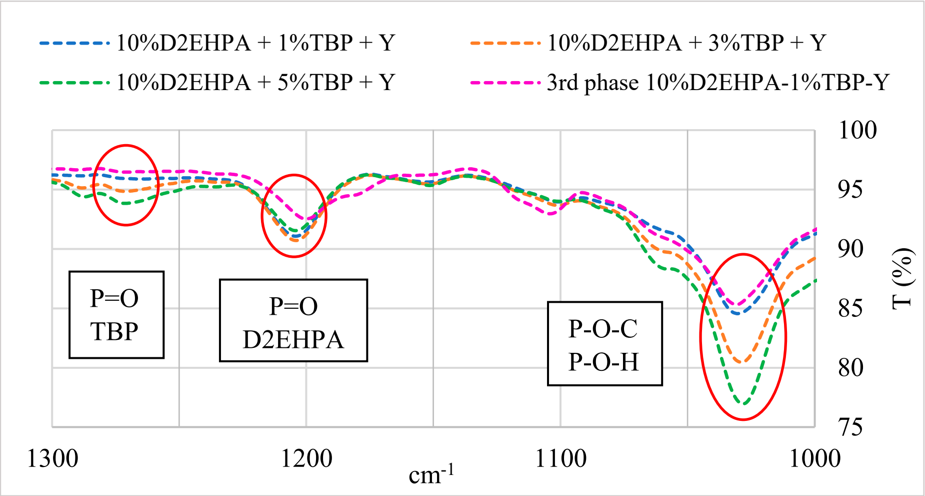

Figure S3. FT-IR spectra of 10 v/v% D2EHPA in kerosene with 1, 3 and 5 v/v% TBP equilibrated with an yttrium solution and a third phase formed in 10 v/v% D2EHPA with 1 v/v% TBP in kerosene.

As can be seen in Figure S4, the loading of the organic phase with Y(III) shifted the P=O band to lower wavelengths due to the coordination with the metal (1% 1-decanol: 1203.94 cm^{-1}; 3% 1-decanol: 1203.18 cm^{-1}; 5% 1-decanol: 1203.46 cm^{-1}; 3rd phase 1% decanol: 1201.08 cm^{-1}). The P-O-C/P-O-H band shifted to slightly lower wavelengths and decreased in intensity due to the release of hydrogen by metal complexation (1% 1-decanol: 1031.76 cm^{-1} – 0.07 A; 3% 1-decanol: 1031.83 cm^{-1} - 0.07 A; 5% 1-decanol: 1031.89 cm^{-1} - 0.08 A; 3rd phase 1% 1-decanol: 1032.43 cm^{-1}, 0.07 A).

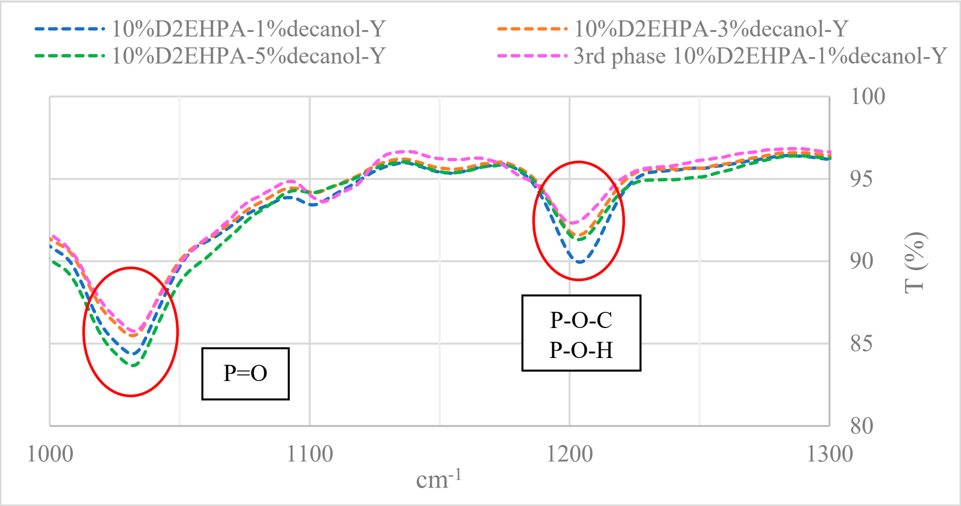

Figure S4. FT-IR spectra of 10 v/v% D2EHPA in kerosene with 1, 3 and 5 v/v% 1-decanol equilibrated with an yttrium solution and a third phase formed in 10 v/v% D2EHPA with 1 v/v% 1-decanol in kerosene.
